# Porous BiVO_4_/Boron-Doped Diamond Heterojunction Photoanode with Enhanced Photoelectrochemical Activity

**DOI:** 10.3390/molecules27165218

**Published:** 2022-08-16

**Authors:** Jiangtao Huang, Aiyun Meng, Zongyan Zhang, Guanjie Ma, Yuhao Long, Xingyu Li, Peigang Han, Bin He

**Affiliations:** 1 College of New Materials and New Energies, Shenzhen Technology University, Shenzhen 518118, China; 2 College of Applied Technology, Shenzhen University, Shenzhen 518118, China; 3 University Engineering Research Center of Crystal Growth and Applications of Guangdong Province, Shenzhen Technology University, Shenzhen 518118, China

**Keywords:** photoelectrochemical (PEC), bismuth vanadate (BiVO_4_), boron-doped diamond (BDD), p-n heterojunction, water splitting, tetracycline hydrochloride (TCH) degradation

## Abstract

Constructing heterojunction is an attractive strategy for promoting photoelectrochemical (PEC) performance in water splitting and organic pollutant degradation. Herein, a novel porous BiVO_4_/Boron-doped Diamond (BiVO_4_/BDD) heterojunction photoanode containing masses of ultra-micro electrodes was successfully fabricated with an n-type BiVO_4_ film coated on a p-type BDD substrate by magnetron sputtering (MS). The surface structures of BiVO_4_ could be adjusted by changing the duration of deposition (T_d_). The morphologies, phase structures, electronic structures, and chemical compositions of the photoanodes were systematically characterized and analyzed. The best PEC activity with the highest current density of 1.8 mA/cm^2^ at 1.23 V_RHE_ was achieved when T_d_ was 30 min, and the sample showed the highest degradation efficiency towards tetracycline hydrochloride degradation (TCH) as well. The enhanced PEC performance was ascribed to the excellent charge transport efficiency as well as a lower carrier recombination rate, which benefited from the formation of BiVO_4_/BDD ultra-micro p-n heterojunction photoelectrodes and the porous structures of BiVO_4_. These novel photoanodes were expected to be employed in the practical PEC applications of energy regeneration and environmental management in the future.

## 1. Introduction

Photoelectrochemical (PEC) catalysis has received much attention in the past decades since it is an efficient way to utilize sufficient solar energy to exploit clean hydrogen energy and treatment environmental hazards [1,2]. The key to improve the PEC efficiency is to develop highly efficient semiconductor photoelectrode materials. Construction of heterojunction photoanodes have been considered to be one of the most promising strategies and widely used in PEC applications. Thus, p-n heterojunction photoanodes formed by combining p-type semiconductors and n-type semiconductors have received more and more attentions [3]. Recently, a novel p-n heterojunction photoanode fabricated by forming an n-type photocatalyst on the surface of a p-type Boron-doped diamond (BDD) has been fabricated and demonstrated excellent PEC activity [4,5]. For these photoanodes, a large number of photogenerated electron-hole pairs generated in the n-type photocatalyst under light irradiation and the photogenerated electrons in the n-type material migrate to the hole-rich of BDD by a driven force from light irradiation and forward bias. At the same time, the holes injected from p-type BDD and generated in the n-type material transfer to an electrolyte, thus achieving an oxidation reaction at the interface of the n-type material and electrolyte directly. Note that the p-type BDD acts as a promising electrode due to its excellent chemical and physical robustness and high thermal and electrical conductivity [6]. In addition, its wide potential window supplies a sufficient bias voltage, and the hydroxyl radicals (•OH) are more likely to be generated on the BDD surface, which are beneficial to catalytic reaction [4,7].

Up to now, TiO_2_/BDD [5,8] and ZnO/BDD [9] heterojunction photoanodes have been successfully fabricated and have exhibited the advantages in practical applications of PEC water splitting and organic pollutant degradation. However, their PEC activities are still limited by the low light absorption efficiency owing to the wide band gap (E_g_) of TiO_2_ and ZnO. In our previous work, the N-doped TiO_2_ could narrow the E_g_ and enlarge the light response to improve the PEC performance [10]. In addition, the structure engineering such as mesoporous TiO_2_/BDD [11], nanostructured TiO_2_/BDD [12], patterned TiO_2_/BDD [4], and 3D Macro−Mesoporous TiO_2_/SnO_2_/BDD [13] heterojunction photoanodes have also been adopted to achieve an enhanced photoelectrocatalytic performance due to their large electroactive surface area, improved light absorption, and efficient substance transport. However, the higher current efficiency is still expected for their practical applications since. Therefore, it is highly desirable to explore the new p-n heterostructure photoanode with an enhanced PEC performance.

The n-type bismuth vanadate (BiVO_4_) is a promising photocatalyst which has been widely investigated for practical applications of organic pollutant degradation and water splitting due to its the advantages including narrow band gap (~2.4 eV), low cost, suitable position of conduction and valence bands, and excellent photoelectric stability [14,15]. In the past decades, BiVO_4_-based photoanodes such as NiO/BiVO_4_ [16,17], MoS_2_/BiVO_4_ [18], FeCoO_x_/BiVO_4_ [19], NiS/BiVO_4_ [20], Cu_2_O/BiVO_4_ [21], CoO/BiVO_4_ [22] and BiOI/BiVO_4_ [23] have been synthesized to obtain an excellent current efficiency owing to an effective carrier separation benefited from the driving force of a built-in electric field formed at the p-n junction [24,25]. However, the photoanodes as above mentioned are generally synthesized by a chemical method, which shows several drawbacks such as small scale, poor recyclability, and low-efficiency production. Moreover, compared with the BDD, the p-type semiconductors such as NiO, MoS_2_, and Cu_2_O always exhibit an extremely poor chemical and physical robustness, and there is no report on BiVO_4_/BDD photoanodes as well. Therefore, a novel heterojunction photoanode with porous structures is proposed by taking the advantages of the n-type BiVO_4_ and the p-type BDD, and further explore the interface charge migration mechanism to enhance the charge transport efficiency and promote the PEC activity.

In this work, a series of porous BiVO_4_/BDD heterojunction photoanodes were fabricated by growing BiVO_4_ films on BDD films. Herein, the BiVO_4_ films and the BDD films were prepared by a magnetron sputtering (MS) and hot filament chemical vapor deposition (HFVCD) method, respectively. The BiVO_4_/BDD heterojunction photoanodes with different thickness of BiVO_4_ films were controlled by the duration of deposition (T_d_) of BiVO_4_. The as-prepared heterojunction photoanodes were systematically characterized to discuss their enhanced PEC performance and the possible mechanism.

## 2. Results and Discussion

### 2.1. Morphological Characterization

**Figure 1:**
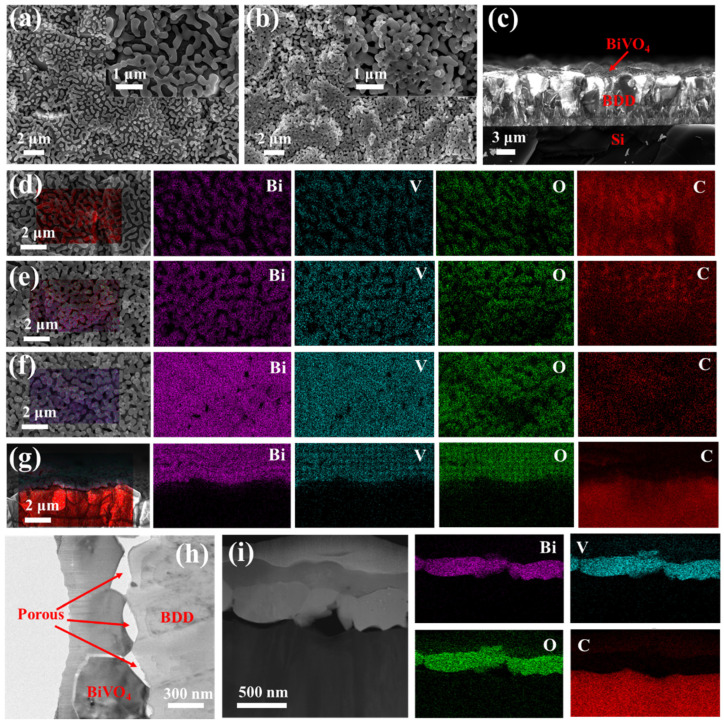
SEM images (top view) and enlarged SEM images (inset) of the BiVO_4_/BDD heterojunction photoanodes for the (**a**) M15 and (**b**) M30; (**c**) SEM image (cross-sectional view) the BiVO_4_/BDD heterojunction photoanode for the M30; Elemental mapping (top view) the BiVO_4_/BDD heterojunction photoanodes for the (**d**) M15, (**e**) M45, and (**f**) M75; (**g**) Elemental mapping (cross-sectional view) the BiVO_4_/BDD heterojunction photoanode for the M45; (**h**) HRTEM image and (**i**) elemental mapping (enlarged cross-sectional view) of the M30.

The series of porous BiVO_4_/BDD heterojunction photoanodes were denoted as M15, M30, M45, M60, and M75. The preparation details of samples were demonstrated in experimental sections. A scanning electron microscopy (SEM) and a high-resolution transmission electron microscopy (HRTEM) equipped with an EDS detector were conducted to observe the morphologies and element mapping of the BiVO_4_/BDD heterojunction photoanodes. As shown in Figure 1a–c, high-quality BiVO_4_ films with grain size in the hundreds of nanometers range are well-dispersed on the dense BDD. The SEM and enlarged SEM images show that the BiVO_4_ films with porous structures partially disperse and then fully overlay on the BDD with the prolonged the T_d_, indicating an increasing thickness of BiVO_4_ films (Figure S1). These porous structures can effectively shorten the hole transport distance and reduce the recombination of photogenerated carriers. Moreover, the BDD partially covered by the porous BiVO_4_ film acts as an ultra-micro electrode to catalyze the PEC reactions. Note that, the amount of the ultra-micro electrodes highly depends on the T_d_ of the BiVO_4_. The elemental mapping results double confirm that masses of ultra-micro electrodes formed on the BDD when the T_d_ is less than 45 min, and the element signal also suggests that a longer T_d_ leads to a thicker BiVO_4_ film (Figure 1d–f). Figure 1g further demonstrates that the BiVO_4_/BDD heterojunction photoanode is fabricated by employing the BiVO_4_ on the BDD film. In addition, a HRTEM image and the element mapping further reveal that the grain size of BiVO_4_ is hundreds of nanometers. Interestingly, the porous structures also present at the interface between the BiVO_4_ and the BDD, which can provide a favorable channel for substance transport (Figure 1h,i). The above results indicate that a series of porous BiVO_4_/BDD heterojunction photoanodes were successfully synthesized. The large grain size, ultra-micro electrodes, and porous structures are beneficial for electrolyte diffusion, charge carrier transfer, and transport, which would effectively promote their PEC activities [4,26].

### 2.2. Crystal Phase and Element Composition

**Figure 2:**
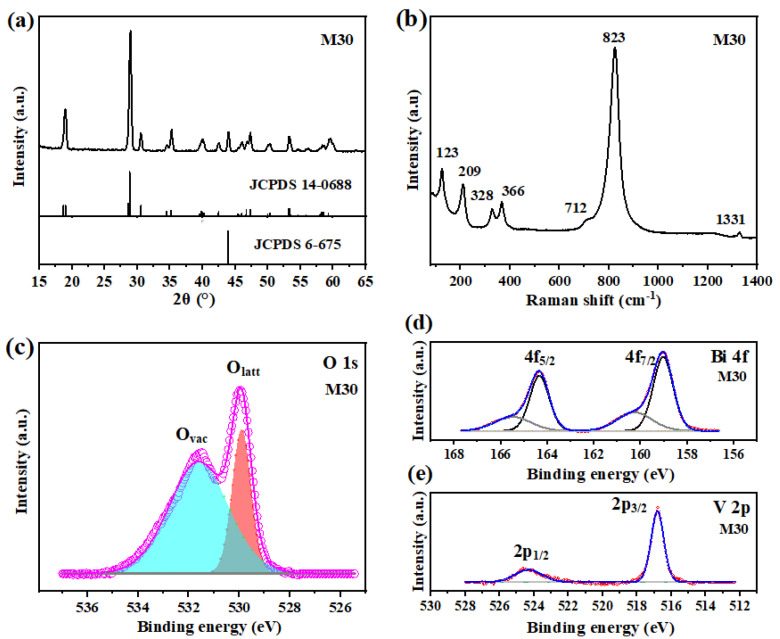
(**a**) XRD patterns, (**b**) Raman spectra, XPS spectra of (**c**) O 1s, (**d**) Bi 4f, and (**e**) V 2p for the M30 BiVO_4_/BDD heterojunction photoanode.

The crystalline structure of M30 BiVO_4_/BDD heterojunction photoanode was investigated by X-ray Diffractometry (XRD) spectroscopy. As shown in Figure 2a, a strong diffraction peak at 2θ of 44° is well matched with (111) plane of diamond (JCPDS: 6-675) [27]. Moreover, the other characteristic peaks are indexed to monoclinic BiVO_4_ (JCPDS: 14-0688) [28], which the main peaks at 2θ of 35.2°, 30.6°, 28.9°, and 18.9° correspond to the planes of (002), (040), (121), and (011), respectively. The XRD data suggests that the as-prepared heterojunction photoanode is formed by high-quality BiVO_4_ and BDD films, which corresponds well to the SEM and HRTEM images (Figure 1). The typical vibrational modes related to the phase structures and defects were easily identified by the Raman spectroscopy (Figure 2b). A characteristic peak at Raman shift of 1331 cm^−1^ corresponds to the crystalline diamond [29]. Other observed Raman shifts at 123 and 209, 328 and 366, 712 and 823 cm^−1^ highly correspond to the monoclinic BiVO_4_ [30], which are assigned to the translation and the rotation external mode, the antisymmetric and the symmetric bending mode, the antisymmetric and the symmetric stretching mode of the VO_4_ tetrahedra, respectively. The monoclinic BiVO_4_ is considered to have the best catalytic activity in photocatalytic applications [31]. In addition, the most intense band at 823 cm^−1^ shows a slight redshift compared with the pristine BiVO_4_ (Raman shift: 829 cm^−1^) reported from literature [32], which may be associated with the formation of defects such as oxygen vacancies [33,34], and it is also confirmed by X-ray photoelectron spectrometer (XPS) (Figure 2c). The peaks at 531.6 eV and 529.9 eV are attributed to oxygen vacancy (O_vac_) and oxygen lattice (O_latt_) existed in BiVO_4_, respectively [35]. Oxygen vacancies that act as electron donors can increase the majority carrier concentration and promote the charge separation efficiency, resulting in a positive significance on the PEC activities [36,37]. The chemical states of Bi 4f and V 2p were also investigated by the XPS. For the Bi 4f high resolution spectrum (Figure 2d), the main peaks at the binding energy of 164.3 eV and 159.0 eV are assigned to Bi^3+^ and the other small peaks at a higher binding energy of 165.5 eV and 160.3 eV can be ascribed to Bi^5+^ in BiVO_4_ [38,39]. The peaks at 516.8 eV and 524.3 eV correspond to V^5+^ existed in BiVO_4_ (Figure 2e).

### 2.3. Electronic Structures

**Figure 3:**
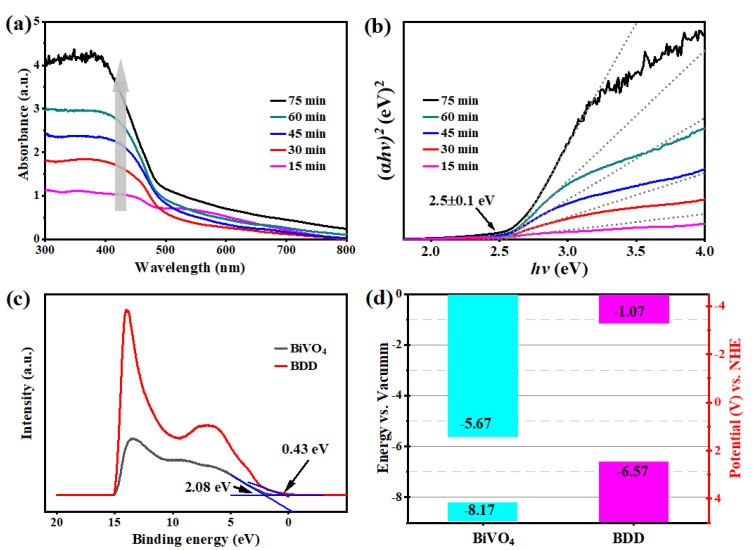
(**a**) The UV-VIS absorbance curves and (**b**) Tauc plots of the BiVO_4_ films with different T_d_; (**c**) UPS spectra for the BiVO_4_ film and the BDD film; (**d**) Energy band positions of the BiVO_4_ and the BDD in reference to vacuum level and NHE.

The absorbance curves of BiVO_4_ films grown on the glass chips with different T_d_ were obtained by a UV-VIS spectrometer (Figure 3a). Obviously, a longer T_d_ of the BiVO_4_ films corresponds to a stronger light absorption. In addition, all the BiVO_4_ films exhibit an absorption edge up to 500 nm, indicating that the solar light response range has been extended to the visible light region, which corresponds to the narrow band gap of 2.5 eV (Figure 3b). Herein, the E_g_ was determined by the Tauc plot based on the following Equation (1) [40]:

(1) $$\left(\alpha h\nu \right)=A{\left(h\nu -E_{g}\right)}^{n/2}$$

where h is the Planck’s constant, A is the constants, ν is the frequency of the incident light, α is the absorption coefficient, and n is 1 as BiVO_4_ is a kind of direct-gap semiconductor.

To further evaluate the electronic structure, an ultraviolet photoelectron spectroscopy (UPS) was conducted to determine the band edge positions of BDD and BiVO_4_. As shown in Figure 3c, the valence band maximum (E_VB_) of the BiVO_4_ and BDD is 2.08 eV and 0.43 eV, respectively. Accordingly, the work function (Φ) is calculated by Φ = hν − E_cutoff_, where hν and E_cutoff_ correspond to the excitation source energy and the secondary electron cutoff, respectively [41]. Moreover, the work functions of BDD and BiVO_4_ are also calculated by Device Studio software as shown in Figure S2. Both experimental and theoretical results indicated that the Fermi level (E_F_) of BDD is lower than that of BiVO_4_, which is beneficial to the formation of the p-n heterojunction and the built-in electric field directed from BiVO_4_ to BDD. In addition, the conduction band minimum (E_CB_) is also obtained by E_CB_ = E_VB_−E_g_. Consequently, the energy values and band gap for BiVO_4_ and BDD are listed in Table S1 and the energy band diagrams of BDD and BiVO_4_ are also schematically drawn in Figure 3d.

**Figure 4:**
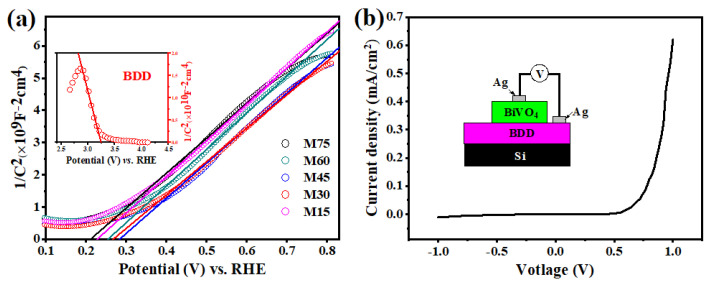
(**a**) The Mott-Schottky plots for the BiVO_4_/BDD heterojunction photoanodes and (inset) the BDD; (**b**) The current-voltage curve of the BiVO_4_/BDD heterojunction photoanode and (inset) the schematic diagram of the corresponding illustration.

The Mott-Schottky plots confirm that the BDD and the BiVO_4_ are p-type and n-type semiconductor, respectively (Figure 4a). The current-voltage curve also demonstrates that a p-n heterojunction is successfully formed at the interface of the BiVO_4_ and BDD (Figure 4b). What is more, the flat band potential (E_fb_) and carrier density (N_D_/N_A_) of BiVO_4_ and BDD were calculated by Equations (2) and (3) [42]:

(2) $$\frac{1}{C^{2}}=\frac{2}{A^{2}{e\varepsilon \varepsilon }_{0}N_{D}}\left(E-E_{\mathrm{fb}}-\frac{{\mathrm{Tk}}_{B}}{e}\right)\;$$

(3) $$N_{D}\left(N_{A}\right)=\left(\frac{2}{A^{2}{e\varepsilon \varepsilon }_{0}}\right){\left[\frac{d\left(\frac{1}{C^{2}}\right)}{\mathrm{dE}}\right]}^{-1}$$

where e and ε_0_ are constants corresponding to the electron charge and the vacuum permittivity, and ε is 68 and 5.6 for the BiVO_4_ and BDD film, respectively [5,43]. The magnitude of N_D_ of BiVO_4_ and N_A_ of BDD is 10^18^, and the E_fb_ of BiVO_4_ and BDD is around 0.25 V_HRE_ and 3.24 V_RHE_, respectively, which are close to reported values [44,45].

### 2.4. Photoelectrochemical and Electrochemical Performance

**Figure 5:**
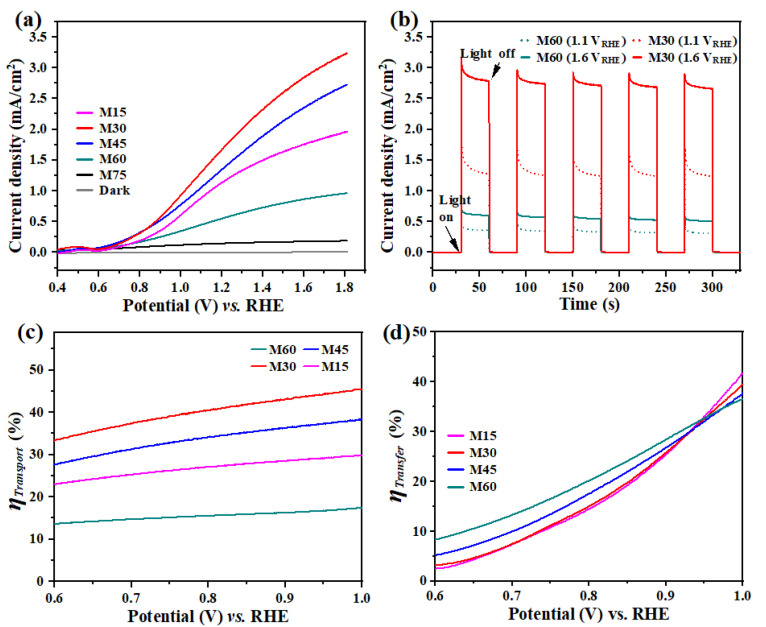
(**a**) Linear sweep voltammogram (LSVs) for the M15-M75 measured in 0.1 M Na_2_SO_4_ under AM 1.5 irradiation and in the dark; (**b**) Variation of the current densities of the M30 and M60 under AM 1.5 irradiation at 1.1 V_RHE_ and 1.6 V_RHE_; (**c**) The calculated η_transport_ and (**d**) η_transfer_ of the M15-M60.

The PEC performance of the BiVO_4_/BDD heterojunction photoanodes was evaluated in 0.1 M Na_2_SO_4_ aqueous solution. Under AM 1.5 irradiation, the current densities of the BiVO_4_/BDD heterojunction photoanodes markedly increase with an applied DC potential from 0.4 V_RHE_ to 1.8 V_RHE_ (Figure 5a), and the detected current densities are also stable and reproducible (Figure 5b). Note that the current densities highly depend on the T_d_ of BiVO_4_ films. The M30 with the T_d_ of 30 min shows the best current density of 1.8 mA/cm^2^ at 1.23 V_RHE_, which is much higher than that of other photoanodes. The optimized current density is also comparable with or better than that of previously reported BiVO_4_ films fabricated by similar method [35,46].

To better understand fundamental processes occurring on these photoanodes, a hole scavenger method was conducted to evaluate the charge transfer and transport properties [47]. Hence, charge transfer efficiency (η_transfer_) at the interface of the electrode and electrolyte and the charge transport efficiency (η_transport_) in the bulk were evaluated by the Equations (4) and (5) [10]:

(4) $$\eta_{\mathrm{transfer}}=\frac{J_{H_{2}O}}{J_{{\mathrm{SO}}_{3}}}$$

(5) $$\eta_{\mathrm{transport}}=\frac{J_{{\mathrm{SO}}_{3}}}{J_{\mathrm{abs}}}$$

where $J_{H_{2}O}$ is the current density measured in 0.1 M Na_2_SO_4_ under AM 1.5 irradiation (Figure 5a), $J_{{\mathrm{SO}}_{3}}$ is the current density measured in 0.1 M Na_2_SO_4_ containing 0.2 M Na_2_SO_3_ hole scavenger under AM 1.5 irradiation (Figure S3a), J_abs_ is theoretical current density, which is calculated by the maximum photocurrent density (Figure S3b) and the light harvesting efficiency (LHE) (Figure S4). As shown in Figure 5c, with the increasing T_d_ of BiVO_4_ films from 15 min to 30 min, the charge transport efficiency is also significantly improved. However, further increasing T_d_ induced an obvious decrease of the charge transport efficiency. Particularly, the M30 photoanode shows the highest charge transport efficiency, which corresponds to its excellent current density. In addition, the charge transfer efficiencies for M15–M60 heterojunction photoanodes are shown in Figure 5d. The results indicate that the T_d_ of BiVO_4_ films is not strongly responsible for the charge transfer efficiency.

**Figure 6:**
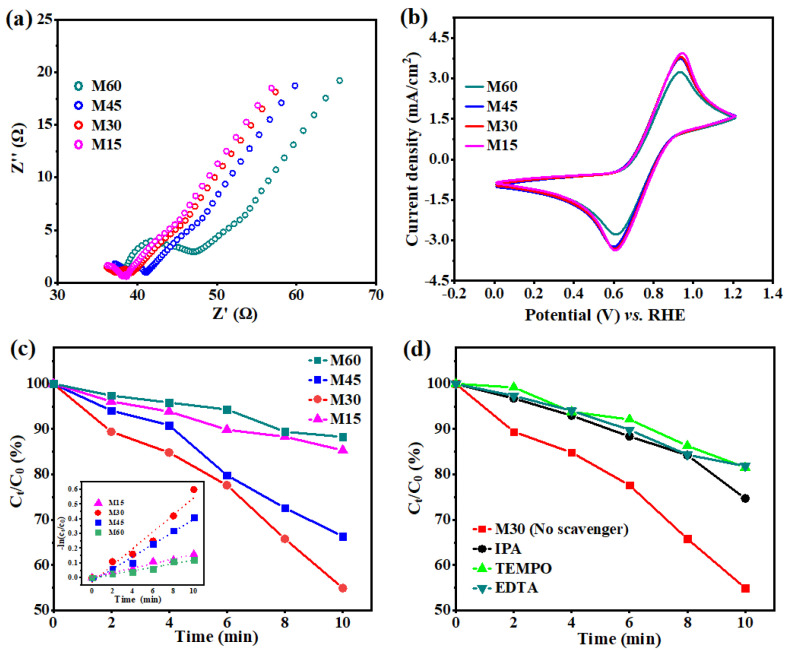
(**a**) EIS Nyquist plots and (**b**) CV curves for the M15-M60; (**c**) TCH degradation and (inset) kinetic curves of TCH degradation for the M15-M60; (**d**) TCH degradation for the M30 with different scavengers.

The electrochemical impedance spectroscopy (EIS) Nyquist plots (Figure 6a) and cyclic voltammetry (CV) curves (Figure 6b) are further demonstrated to evaluate the electrochemical (EC) activities of these samples. The enhanced PEC and EC performance of BiVO_4_/BDD heterojunction photoanodes are ascribed to enhanced charge separation promoted by a large amount of ultra-micro p-n heterojunction photoanodes and porous BiVO_4_ films formed on the surface of BDD (Figure 1).

The practical application of as-prepared photoanodes for PEC degradation of organic pollutants was explored. Tetracycline hydrochloride (TCH) with a concentration of 20 mg/L was selected as a representative substance. As shown in Figure 6c, the TCH removal of the M30 is 45.1% after 10 min, which is much higher than that of other photoanodes (M15: 14.7%, M45: 33.7%, and M60: 11.8%). Moreover, the degradation process also fits a linear first-order kinetics model within 10 min (The inset in the Figure 6c). The rate constants (k) of M15, M30, M45, and M60 are 0.015 min^−1^, 0.057 min^−1^, 0.042 min^−1^, and 0.012min^−1^, respectively. The TCH degradation results are consistent with the current density results as shown in Figure 5a. The TCH degradation results suggest that the M30 exhibits an evidently enhanced PEC degradation activity than that of others photoanodes. In addition, Table 1 summarizes the PEC degradation results of BiVO_4_-based and BDD-based heterojunction photoanodes in recent years, suggesting this novel porous BiVO_4_/BDD photoanode shows a huge potential in treatment of organic pollutants.

To further investigate the active oxidant species during the PEC degradation process, a series of quenching experiments was carried out with kinds of scavengers for the M30. In general, the active oxidant species include superoxide radicals (•O_2_^−^), •OH, and holes (h^+^). Herein, the common scavengers include ethylene diamine tetra-acetic acid (EDTA), isopropyl alcohol (IPA), and 2,2,6,6-tetramethylpiperidine-1-oxyl (TEMPO), which correspond to the h^+^, •OH, and •O_2_^−^, respectively [50]. The quenching experiments results show that the TCH degradation rate of the M30 is significantly limited by the addition of scavengers (Figure 6d). The results indicate that •OH, •O_2_^−^, and h^+^ play an equal contribution during the degradation process. The related degradation mechanism has been proved by the recent literature [1,21]. Furthermore, the degradation products of TCH degradation have been detected by liquid chromatography mass spectrometer (LC-MS), and possible degradation pathways of TCH degradation have been proposed as well (Figure S5). There are various intermediates generated during the decomposition process and the degradation pathway is not unique, which have been reported in some literatures [51,52]. Herein, the TCH degradation is explained as the molecular structure of TCH is gradually destroyed to various small molecular weight (MW) compounds via the reaction with various active oxidant species, and the TCH may further decompose to inorganic ions (NH_4_^+^, NO_3_^−^, H_2_O, and CO_2_) [53].

### 2.5. Possible Mechanism

To further understand the enhanced PEC performance for these novel BiVO_4_/BDD heterojunction photoanodes, the possible mechanism was proposed. In an equilibrium state, a band bending occurs at the interface between the n-type BiVO_4_ and the p-type BDD to maintain the same Fermi level (Figure S6). Note that, a potential barrier formed at the p-n junction of BiVO_4_ and BDD prevents electrons diffusing from BiVO_4_ to BDD as well as holes diffusing in an opposite direction, thus the current density is negligible when driven by no light irradiation or forward bias (Figure 5a).

**Figure 7:**
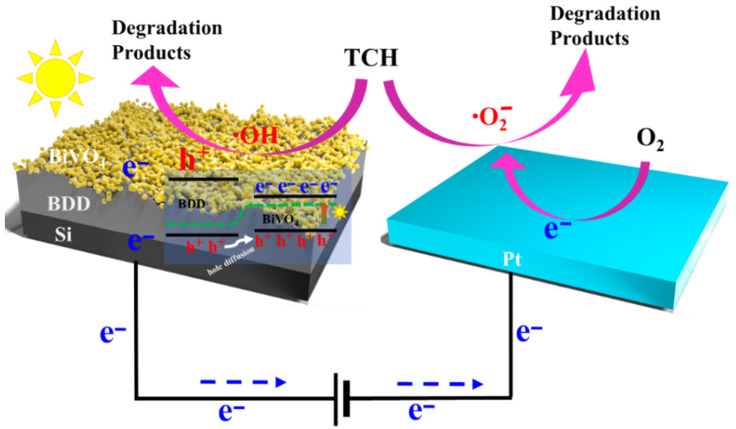
Schematic illustration of the band diagram and PEC degradation occurring at the BiVO_4_/BDD heterojunction photoanode under a forward bias and light irradiation.

However, under light irradiation and additional forward bias, the valence band of BDD containing sufficient hole concentration shifts to equal to or lower than that of BiVO_4_ (Figure 7). Subsequently, the holes inject from BDD to BiVO_4_, and the electrons migrate from BiVO_4_ to BDD as well. Such an efficient charge separation at the interface of BiVO_4_/BDD heterojunction process is driven by light irradiation and additional forward bias [5]. Hence, the holes from the BiVO_4_ enter the solution to complete the oxidation reaction as well as the electrons from the BiVO_4_ migrate to the cathode achieve the reduction reactions, respectively.

Compared with a conventional heterojunction, masses of ultra-micro p-n heterojunction electrodes and porous structures formed on the BDD, which significantly promotes its charge transport efficiency [4,54]. The M30 shows the best PEC performance indicates that an optimized parameter for obtaining the ultra-micro p-n heterojunction is needed. The poor PEC activity for the M75 and M60 is explained as there is no ultra-micro p-n heterojunction electrodes on the BDD. In addition, a thicker BiVO_4_ films on the BDD increases the distance of carrier migration from the bulk to the surface and weakened the charge transport due to the recombination of photogenerated carriers.

## 3. Experimental Section

### 3.1. Synthesis of Materials

The synthesis of porous BiVO_4_/BDD heterojunction photoanodes was illustrated in Figure S7. First, a BDD film was deposited on a conductive silicon (Si) substrate by a HFCVD equipment in a hydrogen/trimethyl borane (CH_4_/H_2_/TMB) gas mixture. Before BDD growth, the fresh Si substrates were seeded with diamond nanoparticle [10]. Then, an amorphous BiVO_4_ film with V rich (V-BiVO_4_) was deposited on the top of BDD film by a MS system with a vanadium (V) target and a BiVO_4_ target in a O_2_/Ar gas mixture. Herein, a series of V-BiVO_4_/BDD composite films with different thickness of V-BiVO_4_ film were obtained by tuning the sputtering time. Subsequently, 0.3 mL of vanadium (V) solution was dropwise added on the as-prepared V-BiVO_4_/BDD composite films, then annealing at 500 ℃ for 120 min at atmospheric condition [35]. Herein, the V solution was prepared by dissolving 10.6 g vanadyl acetylacetonate (VO(acac)_2_) into 200 mL dimethyl sulfoxide (DMSO). Finally, a series of BiVO_4_/BDD heterojunction photoanodes with porous structure were successfully synthesized after removing the excess V_2_O_5_ using 1 M NaOH solution. The as-prepared photoanodes were defined as M15, M30, M45, M60, and M75, which corresponded to the sputtering time of 15 min, 30 min, 45 min, 60 min, and 75 min, respectively. The parameters for BDD (Table S2) and BiVO_4_ (Table S3) growth are shown in supplementary information.

### 3.2. Characterizations

The morphologies and element mapping were characterized by the SEM (Carl Zeiss Microscopy, Jena, Thuringia, Germany) and the HRTEM (FEI, Eindhoven, The Netherlands) equipped with an EDS detector. The crystalline structures and phase structures were identified by the XRD (Panalytical Empyrean, Almeo, The Netherlands) and Raman system (Renishaw inVia, Gloucestershire, UK) equipped with an excitation wavelength of 532 nm. The XPS (Thermo Fisher Scientific, Waltham, MA, USA) test was conducted by using the radiation source of Al Kα to investigate the chemical composition. The UV-VIS spectrophotometer (Shimadzu, Kyoto, Japan) was used to obtain the absorbance curves. The band edge positions were determined by the UPS (Thermo Fisher Scientific, Waltham, MA, USA) with an excitation source of He(I).

### 3.3. PEC and EC Measurements

The PEC and EC performance were evaluated by the CHI 760E electrochemical workstation (CH Instruments Inc., Shanghai, China) under AM 1.5 irradiation (100 mW/cm^2^). Herein, V_RHE_ was calculated by V_RHE_ = V_Ag/AgCl_ + 0.1976 + 0.059 pH, where V_RHE_ and V_Ag/AgCl_ are the potentials versus an RHE and an Ag/AgCl electrode, respectively. The LSV curves were obtained in 0.1 M Na_2_SO_4_ aqueous solution with a scan rate of 30 mV/s. The EIS Nyquist plots were obtained at an open circuit potential with an amplitude of 8 mV at the frequency of 0.1–10,000 Hz. The CV tests were also performed in 0.1 M Na_2_SO_4_ containing 0.01 M K_3_Fe(CN)_6_/K_4_Fe(CN)_6_ with a scan rate of 80 mV/s. Mott-Schottky tests were conducted in 0.1 M Na_2_SO_4_ aqueous solution at a frequency of 1000 Hz. The current–voltage curve was obtained by a Probe Station System. Degradation experimental was conducted with TCH concentration of 20 mg/L in 0.1 M Na_2_SO_4_ under AM 1.5 irradiation at 1 V_RHE_. The products of TCH degradation were identified by LC-MS (Agilent, Palo Alto, Santa Clara, CA, USA).

### 3.4. DFT Calculations

All DFT calculations in the Cambridge sequential total energy package CASTEP code are employed. The interaction between ion core and the valence electron was treated by the norm conserving pseudo potentials. The exchange correlation function was used by the generalized-gradient-approximation (GGA) within PBE functional for diamond and BiVO_4_ bulk calculations. The cutoff energy is set to 580 eV, the k points set to 6 × 6 × 6 and 4 × 4 × 2, respectively. Based on these accuracy settings, the convergence tolerance of energy, maximum force, maximum displacement, and SCF tolerance become 1.0 × 10^−5^ eV/atom, 0.03 eV/Å, 0.01 Å, and 1.0 × 10^−6^ eV/atom, respectively. After geometry optimization, we build BiVO_4_ (001) and B-diamond (111) slabs. To avoid the interaction between repeated slabs, a uniform vacuum width of 15 Å employed. The k points are set to 5 × 5 × 1.

## 4. Conclusions

In summary, a series of porous BiVO_4_/BDD heterojunction photoanodes were successfully fabricated by growing BiVO_4_ films with different thickness on the BDD films. The morphologies, phase structures, electronic structures, and chemical compositions were comprehensively characterized and analyzed by SEM, HRTEM, XRD, Raman, UV-VIS, XPS, and UPS. Moreover, the PEC and EC activities were also systematically discussed. The results indicated that the porous BiVO_4_/BDD heterojunction photoanode with masses of ultra-micro p-n heterojunctions showed an excellent PEC and EC performance. The highest current density was 1.8 mA/cm^2^ at 1.23 V_RHE_, which was achieved by optimizing the parameter of BiVO_4_ growth. The enhanced PEC performance was ascribed to the excellent charge transport efficiency as well as a lower carrier recombination rate, which benefited from a series of porous BiVO_4_ and ultra-micro p-n heterojunction electrodes formed on the BDD. The novel BiVO_4_/BDD heterojunction photoanodes were expected to be employed in the practical PEC application of energy regeneration and environmental management in the future.

## Figures and Tables

**Table 1:** Comparative PEC degradation results of BiVO_4_-based and BDD-based heterojunction photoanodes in recent years.

Photoanodes	Experimental Conditions	Degradation Activity	Years and Ref.
TiO_2_/BDD	Glyphosate (50 mg/L); UVC light lamp (9 W and λ = 254 nm); 5 mA/cm^2^.	Removal: 99.5% (5 h); k = 0.0081 min^−1^	2021 [8]
SnO_2_/Mo: BiVO_4_	TCH (5 mg/L); AM 1.5 G; 1.23 V_RHE._	Removal: 82.1% (120 min); k = 0.00149 min^−1^	2022 [1]
F-BiVO_4_@NiFe-LDH	TCH (20 mg/L); Simulated solar light (100 mW cm^−2^); 0.5 V vs. Ag/AgCl.	Removal: 86% (2 h); k = 0.0156 min^−1^.	2020 [15]
Patterned TiO_2_/BDD	MO (50 mg/L); Simulated solar light (100 mW cm^−2^); 2.5 V vs. Ag/AgCl.	Removal: 100% (4 h).	2020 [4]
BiVO_4_/Ag/Cu_2_O	RhB (5 mg/L); AM 1.5 G; 1.2 V_RHE_.	Removal: 86% (120 min); k = 0.01586 min^−1^.	2022 [21]
WO_3_/BiVO_4_	RhB (5 mg/L); Visible-light; 1.0 V vs. Ag/AgCl.	Removal: 93% (3 h);	2020 [48]
Coral-like WO_3_/BiVO_4_ photoanode	Sulfamethoxazole (20 mg/L); AM 1.5 G; 1.5 V vs. Ag/AgCl.	Removal: 82.1% (120 min)	2022 [49]
Porous BiVO_4_/BDD	TCH (20 mg/L); AM 1.5; 1.0 V_RHE._	Removal: 45.1% (10 min); k = 0.057 min^−1^	This work

## Data Availability

Not applicable.

## Supplementary Materials

The following supporting information can be downloaded at: https://www.mdpi.com/article/10.3390/molecules27165218/s1. The SEM images, LSVs curves, the theoretical current density (J_abs_), LHE, DFT calculations, products of TCH degradation, schematic of the energy band structure for BiVO_4_/BDD heterojunction, and the schematic illustration and parameters for BiVO_4_ and BDD growth. Ref. [55] are cited in the Supplementary Materials.
