# Supplementary material for: Porous BiVO4/Boron-Doped Diamond Heterojunction Photoanode with Enhanced Photoelectrochemical Activity
Source: Molecules. 2022 Aug 16;27(16):5218. doi: 10.3390/molecules27165218 (PMC9415291; doi:10.3390/molecules27165218)
Supplement: Supplementary file 1 [file molecules-27-05218-s001.zip › molecules-1842917 Supplementary update.pdf]

Supplementary

Porous $BiVO_4$/Boron-doped Diamond Heterojunction Photoanode with Enhanced Photoelectrochemical Activity

Jiangtao Huang [1,2,3], Aiyun Meng [1,3,*], Zongyan Zhang [1,3] Guanjie Ma, [1,3], Yuhao Long [1,3], Xingyu Li [1,3], Peigang Han [1,3,*] and Bin He [1,3,*]

[1] College of New Materials and New Energies, Shenzhen Technology University, Shenzhen 518118, China
[2] College of Applied Technology, Shenzhen University, Shenzhen 518118, China
[3] University Engineering Research Center of Crystal Growth and Applications of Guangdong Province, Shenzhen Technology University, Shenzhen 518118, China
* Correspondence: mengaiyun@sztu.edu.cn (A.M.); hanpeigang@sztu.edu.cn (P.H.); hebin@sztu.edu.cn (B.H.)

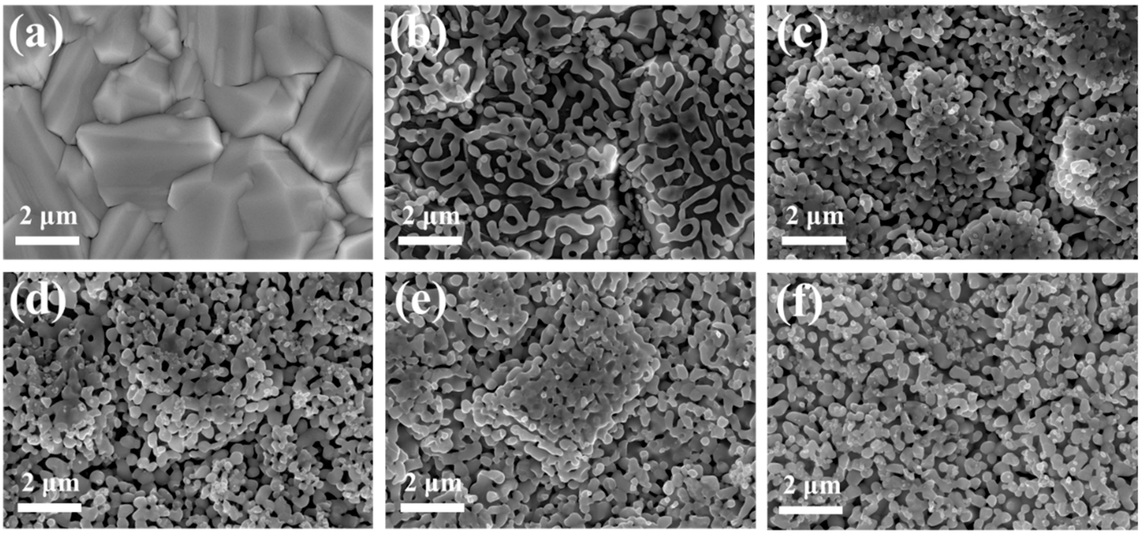

Figure S1. SEM images of $BiVO_4$/BDD heterojunction photoanodes:(a) bare BDD film, (b) M15, (c) M30, (d) M45, (e) M60 and (f) M75, respectively.

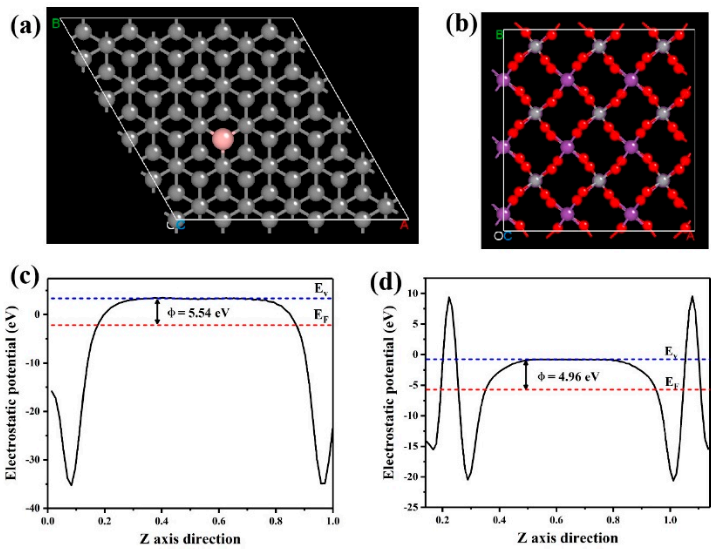

Figure S2 The crystal structures and electrostatic potentials of (a, c) BDD and (b, d) $BiVO_4$ (001) planes. The gray, pink, red, purple and light gray spheres represent for C, B, V, Bi and O atoms. The red and blue dashed lines stand for the Fermi potential and vacuum energy potential, respectively.

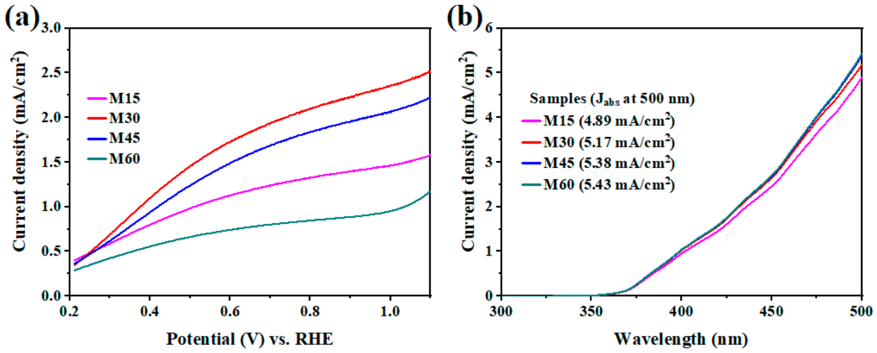

Figure S3. (a) LSVs for the M15 - M60 measured in 0.1 M Na_2SO_4 containing 0.2 M Na_2SO_3 under AM 1.5 irradiation; (b) theoretical current densities calculated based on wavelength of 300 - 500 nm for M15-M60.

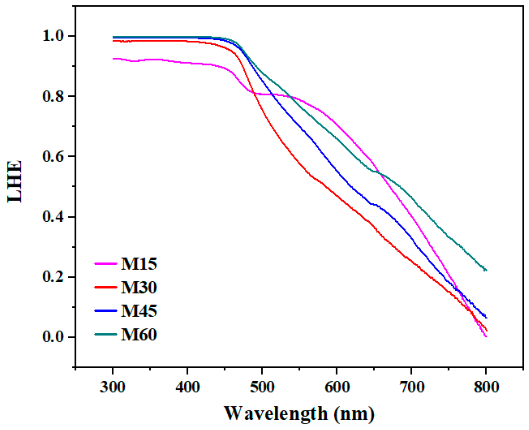

Figure S4. Light harvesting efficiency (LHE) of $BiVO_4$/BDD heterojunction photoanode for the M15 - M60. LHE was obtained based on the equation: LHE = $1-10^{-A(\lambda)}$, where A(λ) is absorbance corresponding to the wavelength (λ) [24].

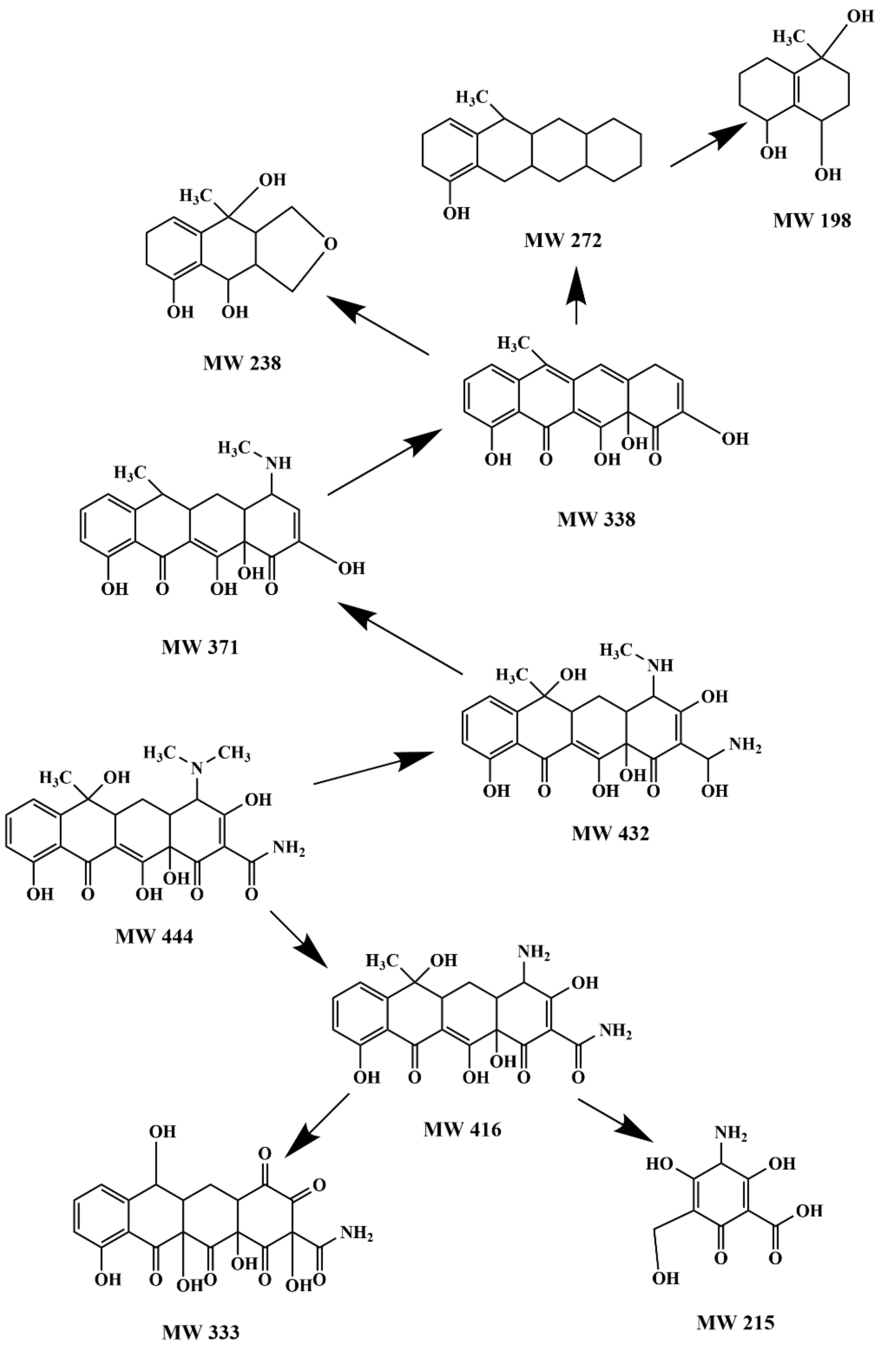

Figure S5. The degradation products and possible degradation pathways of TCH degradation.

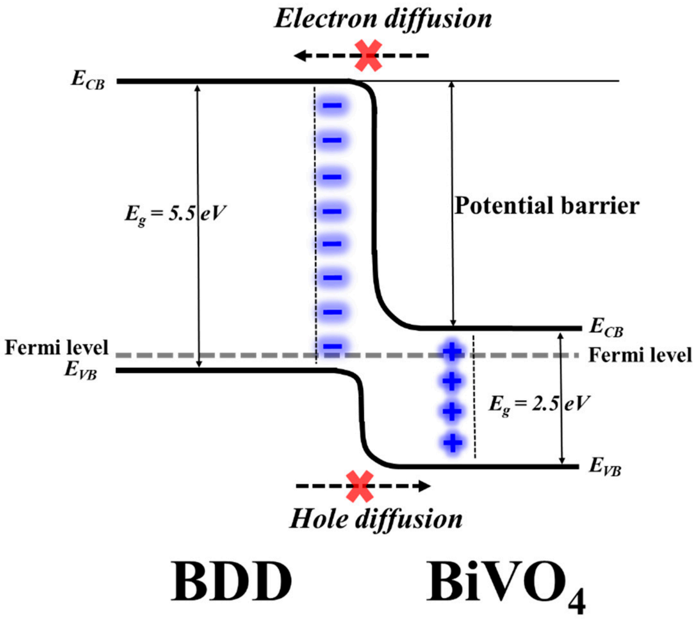

Figure S6. Schematic of the energy band structure of a $BiVO_4$/BDD heterojunction in an equilibrium state.

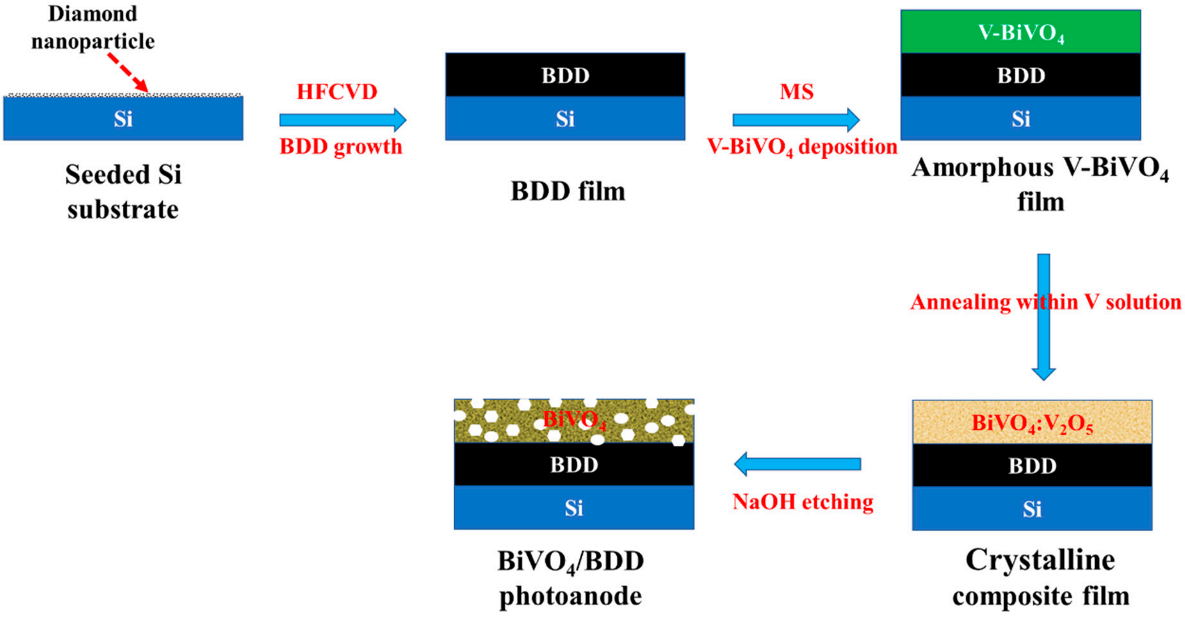

Figure S7. Schematic illustration for the synthesis of porous $BiVO_4$/BDD heterojunction photoanodes.

Table S1. Comparison of energy values and band gap for $BiVO_4$ and BDD.

	$BiVO_4$	BDD
Valence band maximum (E_{VB}) (eV)	-8.17	-6.57
Conduction band minimum (E_{CB}) (eV)	-5.67	-1.07
Work function (Φ) (eV)	6.09	6.14
Band gap (Eg) (eV)	2.5	5.5 [24]

Table S2. Parameters of BDD film growth.

Growth parameters	Values/Unit
Background pressure (Torr)	6.0E-6
Working pressure (Torr)	30
H_2 flow (sccm)	500
CH_4 flow (sccm)	20
TMB flow (99.9% Hydrogen dilution) (sccm)	20
Substrate-to-filament distance (mm)	10
Deposition time (hour)	8
Substrate temperature (°C)	820±15

Table S3. Parameters for $BiVO_4$ films deposition.

	Duration of deposition (T_d) (min)	Flow ratio O_2/Ar (%)	Power of V target (W)	Power of $BiVO_4$ target (W)	Working pressure (Torr)
M15	15				
M30	30				
M45	45	40	250	250	0.015
M60	60				
M75	75				
